# An Athlete’s Diet

**Published:** 1890-08

**Authors:** 


### AN ATHLETE'S DIET.

A well known athlete on being interviewed, gave the following account of his mode of living :

"I always have eaten since I was a boy, plenty of nourishing, generous food, and I am very wide in my choice, eating, as a rule, any good food that tempts my appetite, and that is hearty enough to be easily tempted. For myself, I am not specially fond of what you call made dishes, but prefer food in its plainer forms, which I wash down with copious draughts of light wine. For meats, I eat chiefly mutton and beef, and I use a good deal of bread, of course, being as careful as I can to get the best. My own idea is that so long as you have sound, sweet food, it doesn't make as much difference what kind it is as how you eat it. I am very particular to eat slowly. I eat three times a day. Breakfast is a light or hearty meal, according to how I feel about it at the time. Lunch in the middle of the day is always light, and dinner at 6:30 or 7 is the principal meal of the day. I always take an hour for that. If I haven't an hour to spare at dinner time I put off dinner until I have the time.

"I find, though, that aside from meat and bread, I must have plenty of vegetables. No man can make any kind of an athlete without eating plenty of vegetables. I take all kinds, and pretty much of all fruit, too. Fruits are good. A man can't stay without that kind of food. He has no endurance.

"Yes, I'm Scotch, and I believe in oatmeal, but I don't think you ought to eat too much of it. I have it at breakfast about three times a week. I am fond of milk, too, and am specially careful to drink it slowly. It's excellent food, but it's very bad to drink it fast. I drink wine only at my meals.

"Light wines and tea are my greatest stimulants. I don't drink much coffee, but I do take considerable tea—black tea, always. I never use green—and I take it with sugar and milk, and never take it iced. I don't believe in iced drinks. I don't mean when I say it is my greatest stimulant that I never take any thing stronger. I very seldom do, but sometimes just before a race for instance, I take some brandy ; no malt liquor. That's bad, especially lager. Lager is very bad.

"In training? Well, I make no difference in my diet in training. I only try to keep more regular hours, especially in sleeping. And I take no physic. Physic is bad always. In training, it's fatal."

